# Electron FLASH radiotherapy in vivo studies. A systematic review

**DOI:** 10.3389/fonc.2024.1373453

**Published:** 2024-04-09

**Authors:** Noemi Giannini, Giovanni Gadducci, Taiusha Fuentes, Alessandra Gonnelli, Fabio Di Martino, Paola Puccini, Monica Naso, Francesco Pasqualetti, Simone Capaccioli, Fabiola Paiar

**Affiliations:** ^1^ Department of Translational Research and New Technologies in Medicine and Surgery, University of Pisa, Pisa, Tuscany, Italy; ^2^ Centro Pisano Multidisciplinare Sulla Ricerca e Implementazione Clinica Della Flash Radiotherapy (CPFR), University of Pisa, Pisa, Italy; ^3^ Unit of Medical Physics, Azienda Ospedaliero-Universitaria Pisana, Pisa, Tuscany, Italy; ^4^ National Institute of Nuclear Physics (INFN)-section of Pisa, Pisa, Tuscany, Italy; ^5^ Department of Radiation Oncology, Azienda Ospedaliera Universitaria Pisana, University of Pisa, Pisa, Tuscany, Italy; ^6^ Department of Physics, University of Pisa, Pisa, Tuscany, Italy

**Keywords:** UHDR, electron FLASH, healthy tissue sparing, clinical translation, dosimetry, VHEE, beam parameters, in vivo studies

## Abstract

FLASH-radiotherapy delivers a radiation beam a thousand times faster compared to conventional radiotherapy, reducing radiation damage in healthy tissues with an equivalent tumor response. Although not completely understood, this radiobiological phenomenon has been proved in several animal models with a spectrum of all kinds of particles currently used in contemporary radiotherapy, especially electrons. However, all the research teams have performed FLASH preclinical studies using industrial linear accelerator or LINAC commonly employed in conventional radiotherapy and modified for the delivery of ultra-high-dose-rate (UHDRs). Unfortunately, the delivering and measuring of UHDR beams have been proved not to be completely reliable with such devices. Concerns arise regarding the accuracy of beam monitoring and dosimetry systems. Additionally, this LINAC totally lacks an integrated and dedicated Treatment Planning System (TPS) able to evaluate the internal dose distribution in the case of in vivo experiments. Finally, these devices cannot modify dose-time parameters of the beam relevant to the flash effect, such as average dose rate; dose per pulse; and instantaneous dose rate. This aspect also precludes the exploration of the quantitative relationship with biological phenomena. The dependence on these parameters need to be further investigated. A promising advancement is represented by a new generation of electron LINAC that has successfully overcome some of these technological challenges. In this review, we aim to provide a comprehensive summary of the existing literature on in vivo experiments using electron FLASH radiotherapy and explore the promising clinical perspectives associated with this technology.

## 1 Introduction

Radiotherapy (RT) stands out as one of the most effective anti-cancer treatments used across different tumors types with both curative and palliative intent. The delivery of tumoricidal doses is often associated with severe damage to surrounding normal tissues, often leading to life-threatening toxicities and/or a detrimental impact on the quality of patients’ life (1).

Over the past decades, dose fractionation, image guided radiotherapy (IGRT) and intensity modulated radiotherapy (IMRT) have been implemented to address this challenging problem (2). Furthermore, adaptive radiotherapy (ART) has been used to modify the treatment plan according to the anatomical changes during RT delivery, thereby augmenting its therapeutic ratio (3). However, the efficacy of conventional RT approaches has plateaued, and achieving further improvements in dose conformation would require significant investments in human and technological resources (4).

FLASH-RT, consisting of irradiation with an ultra-high dose-rate (UHDR) (>40 Gy/s) compared to conventional-RT (CONV-RT) (<8Gy/min), emerges as a potentially revolutionary technique (5). Recent animal studies have shown that FLASH-RT can spare normal tissue without compromising its anti-cancer activity. FLASH effect has been highlighted with all kinds of particles currently used in RT (6–8), with a notable emphasis on electrons (9).

This review will delve into the available in vivo studies conducted to date with low energy electrons, which have investigated physical-dosimetric aspects critical for the potential employment of FLASH-RT in clinical practice.

## 2 Methods

An extensive literature review of relevant articles in English language was performed using the databases of PubMed, Scopus, and Google Scholar, and employing the following keywords: FLASH, Radiotherapy, Electron Flash and Ultra-high dose rate (from 1970 until the end of September 2023).

Inclusion criteria were as follows: In vivo experimental studies published in English language from 1970 until the end of September 2023 that investigated the FLASH RT effect using electrons with a focus on toxicity, efficacy, physical aspects and quality assurance.

There were no restrictions on animal models or in the analysis used to study the FLASH effect on tumor or healthy tissue. The tumor’s origin was not a matter of restriction, whether animal or human.

Exclusion criteria were as follows: systematic reviews, meta-analyses, the use of carbon ion, protons or photons, in vitro studies, studies not written in English.

The feasibility of the studies and data extraction were independently evaluated by three reviewers.

**Figure 1:**
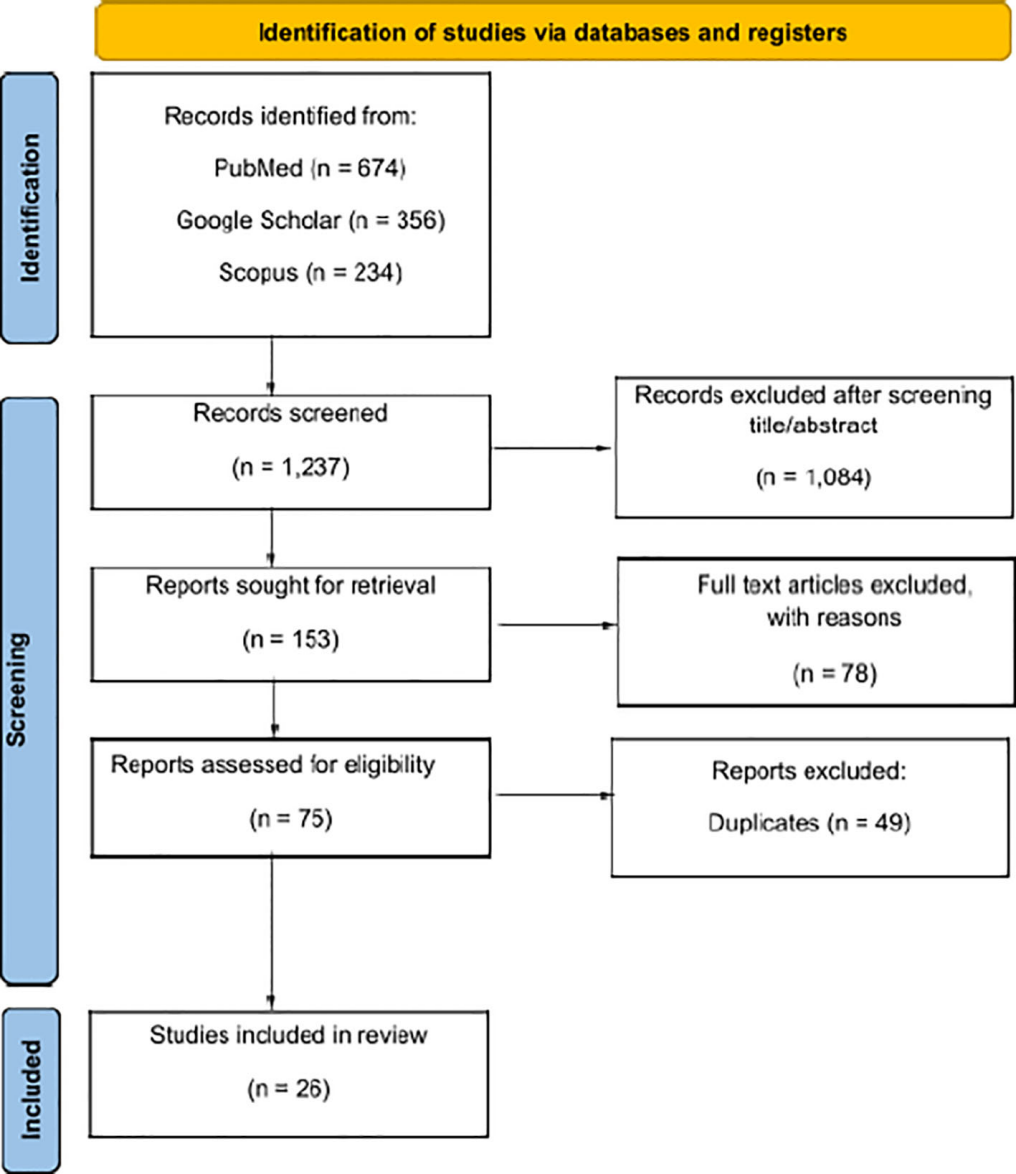
Reporting items for systematic reviews (PRISMA 2020) flowchart (10).

Initially, 1237 articles were identified. After an initial review, 1084 items were excluded, leaving 153 items for full review. Out of these, only 26 articles met the inclusion and exclusion criteria (**Figure 1**). The data, extracted from each article, included as following: name of the first author, type of the study, type of tissue and/or cell, toxicity, and main results. The extracted data were summarized in tables that separated the results obtained from healthy tissue and tumor (**Tables 1**, **2**). Particular emphasis was placed on the modality of irradiation, including the accelerator type, beam energy, physical parameters (FLASH and CONV-RT) and dosimetry (**Table 3**).

**Table 1:** In vivo experiments investigating the FLASH RT in Normal tissues.

Year	Author	Model	Results
1974	Field SB et al. (11)	Rats (Hind feet)	UHDR irradiation results in 30-40% reduction of cutaneous damage compared with CONV-RT.
2014	Favaudon V et al. (12)	C57BL/6J Mice (Lung)	FLASH irradiation prevents lung from radiation induced fibrosis; also spared normal smooth muscle and epithelial cells from acute radiation-induced apoptosis.
2017	Montay-Gruel P et al. (13)	Female C57BL/6J mice (WB)	Preservation of neurogenesis after Flash-RT WB compared to CONV-RT WB.
2017	Loo et al. (14)	Male C57BL/6 Mice (Whole abdomen)	Significantly increased survival after FLASH vs. conventional abdominal irradiation of mice.
2019	Vozenin MC et al. (15)	Pig (Skin)	Acute toxicity after FLASH-RT was limited and transient compared to CONV-RT and late skin fibronecrosis was observed only with CONV-RT.
2019	Venkatesulu BP et al. (16)	Female BALB/c mice (Heart) Male C57BL/6 mice (Spleen) BALB/c mice (Intestine)	The lymphocyte depletion by FLASH-RT was more severe than CONV- RT. FLASH-RT did not have any protective effect against radiation-induced gastrointestinal mucosal lesions.
2019	Montay-Gruel P et al. (17)	Female C57Bl6/J mice (WB)	FLASH did not cause radiation-induced deficits in learning and memory.
2019	Simmons DA et al. (18)	C57BL6/J mice (WB)	Reduced cognitive impairment and associated neurodegeneration were observed with FLASH-RT compared to CONV-RT.
2020	Alaghband Y et al. (19)	C57Bl/6J female mice (WB)	FLASH-RT results in marked neuroprotective properties compared to CONV-RT. FLASH-RT was found to ameliorate radiation-induced cognitive dysfunction, preserve developing and mature neurons, minimize microgliosis and limit the reduction of the plasmatic level of growth hormone.
2020	Soto LA et al. (20)	Female C57BL/6 mice (Skin)	FLASH-RT results in lower incidence and severity of skin toxicity compared to CONV RT.
2020	Montay-Gruel P et al. (21)	C57BL/6Jmice (WB)	FLASH-RT and CONV-RT induces the activation of the complement cascade, but reactive gliosis does not fully develop after FLASH-RT.
2020	Allen BD et al. (22)	Female C57Bl/6J mice (WB)	FLASH-RT reduce levels of apoptosis in the neurogenic regions of the brain and preserves microvasculature integrity in the brain.
2020	Levy K et al. (23)	Female C57BL/6 mice (Whole abdomen)	FLASH-RT produces less DNA damage and/or alters the DNA damage response in intestinal stem cells to enhance crypt regeneration.
2021	Montay-Gruel P et al. (24)	Female Nude Mice (WB)	FLASH-RT was found to significantly spare radiation-induced cognitive deficits in learning and memory in tumor bearing animals after the delivery of large neurotoxic single dose or hypofractionated regimens.
2021	Konradsson E et al. (25)	Canine (Skin)	In general, adverse events observed at the level of irradiated skin with FLASH-RT were mild. Only one case of G3 skin toxicity was observed.
2021	Ruan JL et al. (26)	C3H mice (Whole abdomen)	FLASH RT caused less alteration of the gut microbiota compared to CONV RT, which was shown to be correlated with a reduced intestinal injury.
2021	Chabi S et al. (27)	Mice (TBI)	FLASH-RT TBI reduced functional damage to human blood stem cells.
2022	Rohrer Bley C et al. (28)	Female Goettingen mini pigs (Skin)	No acute toxicity was seen by macroscopic evaluation and subacute toxicity was limited to depilation. However, late skin toxicity was found to occur in a volume-dependent manner.
2023	Allen BD et al. (29)	Female C57Bl/6 mice (WB)	FLASH-RT preserves synaptic connections, structure and density in the hippocampus. Also FLASH-RT does not induce persistent inflammation as observed after CONV-RT.
2023	Limoli CL et al. (30)	C57BL/6J female mice (WB)	A single dose and hypo-fractionated regimens of WB FLASH-RT reduce the adverse cognitive and pathological complications routinely observed after the same fractionation delivered with CONV-RT.

RT, Radiotherapy; CONV, Conventional; UHDR, Ultra High Dose Rate; WB, Whole Brain; TBI, Total Body Irradiation.

**Table 2:** In vivo experiments investigating the FLASH RT on Tumor control.

Year	Author	Model	Results
2014	Favaudon V et al. (12)	Nude mice (Human Breast cancer HBCx-12A and Human Head and Neck cancer HEp-2) C57BL/6J mice (Syngeneic TC-1 Luc+ orthotopic lung tumors)	FLASH is as efficient as CONV in controlling xenografted human tumors and syngeneic orthotopic lung tumors.
2019	Vozenin MC et al. (15)	Cat (T2-T3N0M0 Squamous cell carcinoma)	Tumor growth is under control after a single-dose of FLASH-RT.
2019	Bourhis J et al. (31)	Human (CD30+T-cell cutaneous lymphoma)	Complete and durable tumor response.
2020	Levy K et al. (23)	Female C57BL/6 mice (ID8 ovarian cancer model)	FLASH-RT and CONV-RT had similar efficacy in reducing tumor burden while improving intestinal function.
2021	Konradsson E et al. (25)	Canine (Different superficial solid cancers)	Partial response, complete response or stable disease recorded in 11/13 irradiated tumors.
2021	Kim YE et al. (32)	Male C57BL/6 mice (LLC)	Rapid vascular collapse induced by CONV-RT does not occur by FLASH-RT. CONV RT causes MLC phosphorylation in LLC cancer cells and endothelial cells, leading to vascular collapse in tumors.
2021	Montay-Gruel P et al. (24)	Female Nude Mice H454 (GBM)	FLASH and CONV-RT are iso-efficient in delaying GBM growth.
2021	Chabi S et al. (27)	Mice TBI on humanized model of T-ALL	FLASH-RT and CONV-RT TBI were toxic for normal human hematopoiesis, but only FLASH was able to preserve certain functional properties of human blood stem cells/progenitors.
2022	Rohrer Bley C et al. (28)	Cats with Spontaneous Squamous Cell Carcinoma	Complete tumor remission and all but one cat in each group remained tumor free throughout the follow-up period.
2022	Liljedahl E et al. (33)	Fischer 344 rats (NS1 GMB)	CONV-RT and FLASH were equal anti-tumor efficacy.
2022	Eggold JT et al. (34)	Female C57BL/6 mice (ID8 ovarian cancer model)	FLASH irradiation reduces radiation-induced intestinal injury, it maintains the ability to increase T cell infiltration and reduce immunosuppressive cells in the tumor microenvironment.
2023	Børresen B et al. (35)	Dogs with macroscopic malignant tumors of the oral cavity	FLASH RT was generally effective but with an elevated risk of high grade adverse effects.

RT, Radiotherapy; CONV, Conventional; MLC, myosin light chain; LLC, Lewis lung carcinoma; GBM, glioblastoma; TBI, Total Body Irradiation; T-ALL, T cell acute lymphoblastic leukemia.

**Table 3:** Summary of the physical and dosimetric aspects used for the studies.

Year/Author	Model	Radiation source type	Total dose (Gy)	Dose rate (Gy/s)	Energy (MeV)	Beam parameters	Dosimetry
1974 Field SB et al. (11)	Rats (Hind feet)			FLASH:66.6-83.3 CONV: 0.033	7 MeV		Collector monitors
2014 Favaudon V et al. (12)	C57BL/6J Mice (Lung) Nude mice (Human Breast cancer HBCx-12A) Nude mice (Human Head and Neck cancer HEp-2) C57BL/6J mice (syngeneic orthotopic lung carcinoma)	LINAC	Flash: 15-30 Conv: 15-17 Flash: 17 Conv: 17 Flash: 15-25 Conv: 19.5 Flash: 15-28 Conv: 15-28	FLASH:60 CONV: <0.003	4.5 MeV	<500 ms pulses	Chemical dosimeters of electron pulses: Methyl viologen dosimeter
2017 Montay-Gruel P et al. (13)	Female C57BL/6J mice. (WB)	Oriatron 6e and Kinetron	10	FLASH-RT: >100 CONV-RT: 0.1	6/4.5 MeV	Single 1.0-1.8 μs electron pulse. PRF 10-100 Hz	Ionization chamber corrected for chamber saturation with GafchromicTM EBT3 film with TLD and with Alanine pellets
2017 Loo et al. (14)	Male C57BL/6 Mice (Whole abdomen)	LINAC	10-22	FLASH: 70-210 CONV: 0.05	20 MeV		Gafchromic EBT2 film
2019 Vozenin MC et al. (15)	Pig (Skin) Cat (T2-T3N0M0 Squamous cell carcinoma)	Kinetron and Oriatron 6e	22-34 25-41	FLASH: 300 CONV: 0.083	4.5-6 MeV		Gafchromic EBT3 film and alanine pellets. Dose distribution: CTscan and dose calculation in XiO^®^ treatment planning system
2019 Bourhis J et al. (31)	Human, CD30+T-cell cutaneous lymphoma	Oriatron eRT6	15	FLASH: 166.7	5.6 MeV		Alanine pellets and Gafchromic films
2019 Venkatesulu BP et al. (16)	Female BALB/c mice (Heart) Male C57BL/6 mice (Spleen) BALB/c mice (Intestine)	FLASH: Modified decommissio-ned linear accelerator CONV: True Beam linear accelerator	2Gy x 5 1Gy x 5 16	FLASH: 35 CONV: 0.1	20 MeV	Pulse rate of 180Hz, Pulse length of 4 µs. ADR of 32.6 Gy/s for the 2 × 2 cm^2^ field and ADR of 38.8 Gy/s for the 4 × 4 cm^2^ field.	Gafchromic EBT 3 film and CC04 Farmer chamber, TLD
2019 Montay-Gruel P et al. (17)	Female C57Bl6/J mice (WB)	Oriatron 6e	10/14	FLASH-RT: >100 CONV-RT: 0.1	6 MeV	PRF 100 Hz, Pulse width 1.8µs, No. of pulses: 1, TT 1.8·10^-6^s	TLD
2019 Simmons DA et al. (18)	C57BL6/J mice (WB)	Varian Clinac 21EX.	30	FLASH-RT: 200/300 CONV-RT: 0.13	16/20 MeV	Average of 18 pulses of 2µs, average DPP 1.75 Gy and average intra-pulse dose rate of 8.75x10^5^ Gy/s.	Gafchromic EBT2 films
2020 Soto LA et al. (20)	Female C57BL/6 mice (Skin)		30/40	FLASH-RT: 180 CONV-RT: 0.0747	16 MeV	Frequency 90 Hz, DPP 2.0 Gy, ADR 180 Gy/s, IDR (in 5 μs pulse) 4.0 × 10^5^ Gy/s.	Gafchromic EBT3 film
2020 Fouillade C et al (36)	Females C57BL/6J mice (Lung)	Kinetron	5.2 ± 0.2	>20	4.5 MeV		Gafchromic EBT3 film. For FLASH-RT, the films were calibrated with reference to the methyl viologen dosimeter
2020 Alaghband Y et al. (19)	C57Bl/6J female mice	Oriatron 6e	8	FLASH-RT: 4.4×10^6^ CONV-RT: 0.1		PRF 100 Hz, Pulse Width 1.8μs, No. of Pulses: 1, TT 1.8×10^−6^ s	Solid water phantom, positioned behind a EBT3 Gafchromic films
2020 Montay-Gruel P et al. (21)	C57BL/6Jmice (WB)	eRT6/Oriatron	10	FLASH-RT: 5.6 ×10^6^ CONV-RT: 0.1	6 MeV	1–10 pulses of 1.8 μs, Frequency 100 Hz, TT 1.8x10^6^ s.	
2020 Allen BD et al. (22)	Female C57Bl/6J mice	eRT6/Oriatron	10/25	FLASH-RT: 5.6x10^6^ e 2.5x10^3^ CONV-RT: 0.09	6MeV	PRF 100 Hz, Pulse width 1.8 μs, No. of pulses 1-2, TT 1.8x10^6^ s, Dm 5.6-6.9x10^6^ IDR 5.6-6.9x10^6^	
2020 Levy K et al. (23)	Female C57BL/6 mice (Whole abdomen) Female C57BL/6 mice (ID8 ovarian cancer model)	Modified linear accelerator.	14/16	FLASH-RT: 216 CONV-RT: 0.079	16 MeV	2Gy/pulse	EBT3 Gafchromic film
2021 Konradsson E et al. (25)	Canine (Different superficial solid cancers) Canine (Skin)	Clinical Elekta Precise linear accelerator	15/35	400-500	10 MeV	3.5 μs pulses at a PRF of 200 Hz, 7-16 pulses corresponding to a total TT ranging from 30 ms to 75ms	Gafchromic EBT-XD film
2021 Kim YE et al. (32)	Male C57BL/6 mice (LLC)	Varian 21EX, Electron LINAC	15	FLASH-RT: 352.1 + 4.0 CONV-RT: 0.060 + 0.001	16 MeV		Gafchromic film dosimetry (EBT3 film; Ashland Inc, Covington, KY)
2021 Montay-Gruel P et al. (24)	Female Nude Mice (WB) Female Nude Mice H454 (GBM)	eRT6/Oriatron	10/30 4x3.5 Gy, 2x7 Gy, 3x10 Gy Single- dose: 10/14/25	FLASH-RT: > 10^6^ CONV-RT: 0.1	6 MeV	PRF 100 Hz, Pulse width 1.8 μs, No. of pulses 1-2, TT 1.8x10^6^ s, IDR 5.6-6.9x10^6^	
2021 Ruan JL et al. (26)	C3H mice (Whole abdomen)	LINAC	7.5/20	Single pulse FLASH-RT: 2-6×10^6^ CONV-RT: 0.25	6 MeV	No. of pulses of the dose delivery: 1-300 for 11.2 Gy and 1-1250 for 12.5 Gy	Gafchromic EBT-XD film
2021 Chabi S et al. (27)	Mice. (TBI) Mice. TBI, on humanized model of T-ALL	eRT6/Oriatron	4	FLASH-RT: 200 CONV-RT: <0.072	6 MeV	PRF 100Hz, Pulse width1.8 μs, No. of pulses: 3, TT 0.02s, IDR 7.4 × 10^5^Gy/s	TLD
2022 Liljedahl E et al. (33)	Fischer 344 rats (NS1 GMB)	Elekta Precise, Elekta AB.	8x2 Gy 12.5x2Gy	FLASH-RT: >90 CONV-RT 0.13	10 MeV	ADR >90 Gy/s, 3 Gy/pulse, IDR 0.85x10^6^Gy/s, total TT ≤ 170 ms	Gafchromic EBT3 film
2022 Eggold JT et al. (34)	Female C57BL/6 mice (ID8 ovarian cancer model)	Configured Varian Trilogy radiotherapy system	14	CONV-RT: 0.126 FLASH-RT: 210	16 MeV	2 Gy/pulse at the entrance surface of the mouse	Gafchromic EBT3 films
2022 Rohrer Bley C et al. (28)	Female Goettingen mini pigs Cats with Spontaneous Squamous Cell Carcinoma	eRT6/Oriatron	31 FLASH-RT: 30 CONV-RT: 10x 4.8	163 FLASH-RT: 6.3 x10^6^ CONV-RT: 0.1	6 MeV 6. 9/12 MeV	IDR 8.61E+05, No. of Pulses 20, Frequency 100 Hz, Pulse Width 1.8µs, TT 190 ms. 30 Gy were delivered in 20ms using 3 pulses Dm 1.500 Gy/s.	Gafchromic EBT-XD film and alanine pellets
2023 Børresen B et al. (35)	Dogs with macroscopic malignant tumors of the oral cavity	Modified Elekta Precise linear accelerator	30/42	≥30-40	10 MeV	DPP: 1.3-2.3 Gy, ADR of ≥115 Gy/s, pulse dose rates ≥3.5*105 Gy/s, TT ≤305 ms.	Gafchromic EBT-XD film
2023 Allen BD et al. (29)	Female C57Bl/6 mice	Oriatron 6e	2/3 x 10Gy	FLASH-RT: 5.6 x10^6^ CONV-RT: 0.09	6 MeV	PRF 100 Hz, Pulse width 1.8 μs, No. of pulses 1, TT 1.8x10^6^ s, Dm 5.6x10^6^ Gy/s. IDR 5.6x10^6^Gy/s	Graphite applicator
2023 Limoli CL et al. (30)	C57BL/6J female mice	Oriatron 6e	10x3Gy	FLASH-RT: 1.6 x10^6^ CONV-RT: 0.09	6 MeV	PRF 100 Hz, Pulse width 1.8 μs, No. of pulses 1.	TLD

RT, Radiotherapy; CONV, Conventional; PRF, pulse repetition frequency; Average dose rate, ADR; WB, Whole Brain; LLC, Lewis lung carcinoma; GBM, glioblastoma; TBI, Total Body Irradiation; DPP, Dose per pulse; T-ALL, T cell acute lymphoblastic leukemia; Dm, Mean dose rate; TLD, Thermo-luminescent dosimeter; TT, Treatment time; IDR, Instantaneous dose rate.

## 3 In vivo studies on the electron FLASH-RT

### 3.1 Hematopoietic system

Total Body irradiation (TBI) is a myeloablative treatment usually delivered in combination with chemotherapy before allogeneic stem cell transplantation (HCT) in patients with acute myeloid leukemia (AML) or acute lymphoid leukemia (ALL) (37). TBI still maintains a key role in conditioning regimens for HCT, although it is burdened by a high rate of 5 years treatment related mortality and serious late adverse events (38). Moreover, the tolerance of standard myeloablative regimens, including TBI, is frequently limited by the age and the presence of concomitant medical illnesses. To enable these patients to obtain allogeneic HCT, it is necessary to de-intensify TBI treatments reducing the total radiation dose delivered (39).

As expected, an increased risk of relapses was observed with reduced-intensity RT regimens, indicating that the benefit on toxicity was burdened by a lower disease control (40–44). Therefore, FLASH- RT has a strong rationale in conditioning schemes for HCT.

Chabi et al. (27) investigated the survival of mice injected with T cell acute lymphoblastic leukemia (T-ALL). Following a week, the animals were exposed to 4 Gy either FLASH-RT or CONV RT, and no discernible overall survival (OS) difference between the two treatment groups was detected. This experiment was also replicated using NSG (NOD scid gamma) immunodeficient mice to study the intrinsic radiosensitivity of T-ALL cells. NSG mice were irradiated 4 weeks after T-ALL injection and euthanized 24h post-RT. T-ALL cells were either cultured in vitro or transplanted in secondary non-irradiated NSG mice.

In vitro, after 7 days from irradiation, the number of T-ALL cells recovered from FLASH RT group was four-fold lower than CONV RT group. In vivo, leukemic cell proliferation was evaluated 7 weeks after transplantation through bone marrow biopsy. FLASH-TBI group showed a lower level of leukemic cells and an improvement of OS compared with CONV-TBI and control group (p=0.0075). Therefore, UHDR-RT showed a higher in vitro and in vivo activity.

This finding demonstrated in vivo and in vitro a greater efficacy of UDHR RT on cancer cells. However, as far as hematopoietic healthy cells are concerned, Venkatesulu et al. (16) demonstrated in 2019 that the ultra-high dose (35Gy/sec) does not spare irradiated immune cells during splenic and cardiac irradiation. In an experiment comparing the effect of FLASH-RT and CONV-RT on circulating lymphocyte count, female BALB/c mice were subjected to cardiac irradiation at a dose of 2 Gy for 5 consecutive days. The reduction in circulating CD3, CD4, CD8 and CD19 cells was comparable in both irradiation modalities. With the FLASH-RT, the decline in lymphocytes was more severe and sustained over time than in the CONV mode. After 24 days post-irradiation, baseline CD3 cells levels were recovered by 100% in mice treated with CONV-RT versus 50% of mice treated with FLASH-RT. The same results were observed with the recovery of CD4, CD8 and CD19 cells. Similarly, a single fraction of 8-Gy in FLASH mode resulted in greater depletion of CD3, CD4, CD8 and CD19 cells than in CONV mode.

These results might be due to the fact that dose rate used was not sufficient in order to induce healthy tissue sparing. Moreover, it is also possible that FLASH effect may affect hematopoietic stem cells (HSC) instead of adult lymphocytes. In fact, Chabi et al. also assessed FLASH RT effect on hematogenic stem cells injected in NSG mice treated with analogous modality and found that UDHR irradiation was able to preserve some level of HSC functionality unlike CONV RT.

### 3.2 Head and neck and skin

Radiation therapy plays a critical role in the optimal management of patients with head and neck cancer (HNC), either as exclusive treatment in stage I or II of disease or in combination with surgery and systemic therapy in locally advanced disease. Despite technological advances, within five years from the end of treatment, approximately one-third of patients with HNSCC experiences a locoregional failure. Tumor progression significantly impacts on both survival and quality of life, resulting in speech and eating difficulties, hindered social interactions, physical deformities, and painful non healing wounds. Furthermore, research has demonstrated that up to 75% of locoregional failures occur in sites treated with high doses, suggesting that HNC often are resistant to conventional RT (45).

Due to this intrinsic radioresistance, it may be beneficial to increase the total dose, mitigating the risk of severe toxicity (mucositis, cutaneous toxicity, such as skin ulceration and subcutaneous fibrosis, dysphagia, odynophagia, loss of taste, xerostomia, oral discomfort, difficulty speaking, osteoradionecrosis, thyroid dysfunction, trismus, sensorineural hearing loss, stenosis and myelitis).

Furthermore, an even more arduous challenge is represented by re-treatment of HNC, as the radiation already absorbed by the tissues limits the dose to be delivered without causing severe damages. The therapeutic window becomes even narrower and complex clinical decision making is required. Primary skin tumors often occur in region of the head and neck, such as nose, ears, eyelids and lips, which represent a difficult challenge for the radiation oncologist to avoid disfiguring chronic side effects (46). Stereotactic radiotherapy is very often exploited in advanced melanoma, which requires high doses per fraction because of its elevated DNA repair capacity. Currently, the unique characteristics of melanoma cells make conventional radiotherapy ineffective in early-stage disease control, as the risk-benefit ratio is not equal to surgery (47).

As far as non-melanoma skin cancers are concerned, radiotherapy may be employed with curative or palliative intent, as either definitive or adjuvant treatment, or with palliative intent. Cutaneous toxicity is the most common side effect, since the particles that cross the skin to reach the target cause damage to the dermo-epidermal cells. This toxicity especially occur when the target is superficial. Early adverse events (AEs) include erythema, wet or dry scaling, hair loss, and ulceration while late AEs, developing after 6 months or more from the end of the treatment, consist of atrophy, fibrosis, telangiectasia, and pigmentation abnormalities (48).

The high occurrence of radiation-induced skin toxicity prompted investigations into the relationship between dose rate and cutaneous tissue damage as early as the 1970-80s by S. B. Field (11) and Inada et al. (49). Mice skin UHDR irradiation (66,6-83,3 Gy/s) resulted in a significant reduction of cutaneous damage compared to CONV RT. Soto et al. (20) obtained similar results (UHDR (180 Gy/s)-CONV RT (0.0747 Gy/s), Doses 30 and 40 Gy) in terms of toxicity.

Vozenin et al. (15) tested electron UHDR on mini pig skin and cats. The total dose delivered ranged from 22 to 34 Gy with both CONV RT (≈5 Gy/min) and FLASH RT modality (≈300 Gy/s). Acute skin toxicity was transient and limited to hair loss with FLASH-RT, while hair follicles were definitely damaged with CONV-RT.

In Phase-I study a UHRD single dose ranging from 25 to 41 Gy was delivered to six cat-patients with locally advanced T2/T3N0M0 squamous-cell-carcinoma of the nasal planum. The results evidenced only mild mucositis and depilation with 16 month-progression free survival of 84%. Afterwards Bley et al. (28) performed a prospective, randomized clinical phase III trial on Cats with T1-T2, N0 carcinomas of the nasal planum. The first arm was treated with an average dose (ADR) rate of 6 Gy/min up to a total dose of 48 Gy in 10 fractions, while the second arm was exposed to an ADR of 1500 Gy/s up to a total dose of 30 Gy in a single fraction. The trial was closed early due to severe late toxicity, since bone necrosis occurred between 9-15 months after RT in 3 of 7 cats of the latter arm and none of the 9 animals of the former arm(p=0.05). This study also tested the sparing capacity of FLASH-RT in mini pigs through the variation of RT volume and found that late skin toxicities were associated with larger fields.

Similar results were published by Konradsson et al. (25) on 10 canine patients with different superficial solid cancers treated with ADR of 400-500 Gy/s up to total RT doses ranging from 15 to 35 Gy. Only one case of G3 skin toxicity was observed. A subsequent prospective study (35) was conducted on 11 dogs affected by a mixed group of malignant oral cancer that were treated with a single fraction of UHDR electron ranging from 30 Gy to 42 Gy. FLASH RT was effective in all dogs, but serious late damage, including osteonecrosis, were sometimes observed. The reconstruction of the treatment plan showed an inhomogeneity of dose distribution with the presence of hotspots outside the target of 42 Gy (120% of target dose prescription). Probably these hotspots caused bone necrosis suggesting the existence of dose value beyond which the FLASH effect is lost.

Finally, FLASH-RT related toxicity was tested on human patients. A 75-year-old patient with multiresistant CD30+ T-cell cutaneous lymphoma was treated at University Hospital of Lausanne with electron FLASH. This patient had previously received repeated treatments with CONV RT using both X-ray and electrons, with G3-4 acute skin reactions. After a single fraction of 15 Gy delivered with UHDR electrons (166,66 Gy/s) a 3.5-cm diameter skin tumor rapidly disappeared with G1 acute skin toxicity and the complete tumor response still persisted after 5 months (31).

### 3.3 Central nervous system

Primary malignant brain tumors remain extremely aggressive cancers. Radiotherapy is pivotal for addressing both adult and pediatric brain tumors, whether primary or metastatic, but a significant challenge of this treatment modality is represented by neurocognitive toxicity with a negative impact on learning, memory, attention, executive skills and mood regulation.

Despite radical surgery followed by high dose radio-chemotherapy treatment (total dose 60 Gy), glioblastoma (GBM) remains one of the most malignant adult tumors with poor prognosis. The reduction of local/in-field recurrences (70-80%) and improvement of outcomes in these patients, especially MGMT non-methylated population, have become the focus of clinical researchers. However, the pursuit of dose-escalation has not yelded robust data due to small patient numbers and population heterogeneity, making it impossible to accurately estimate toxicity (50). Beside acute toxicity, exposure of the brain to ionizing radiation at conventional dose rates is associated with long-term cognitive compromission.

Counteracting the neurological issues resulting from brain irradiation is crucial for improving the well-being of glioblastoma survivors. This need is even more pronounced for individuals afflicted with tumors characterized by a comparatively higher life expectancy, such as low-grade gliomas or meningiomas.

In the pediatric age group, medulloblastoma predominates as the most malignant brain tumor. Chemotherapy and cranio-spinal radiation therapy are combined to develop optimal therapeutic strategies. In view of the curability of this disease, efforts have been made to reduce neurological sequelae without losing the guaranteed effectiveness. For instance, preserving anatomical components, such as the inner ear and the temporal lobes/hippocampus requires a decrease in the boost volume from the posterior fossa to the tumor bed, as explored in the prospective ACNS0331 trial (51). Lastly, thanks to advancements in oncological therapy, the survival of brain metastatic patients has been prolonged too, making it essential to give even more relevance to tolerance aspects. The study of FLASH radiotherapy in these contexts should be emphasized. In the existing scientific literature, various approaches to dose fractionation have been employed, though a majority have focused on single-fraction treatments.

#### 3.3.1 Single dose fraction

The initial UHDR electron study was carried out by Montay-Gruel’s research team in 2017, utilizing tumor-free murine models (13). They explored the potential neuroprotective benefits of FLASH-RT by employing the “Novel Object Recognition” test, conducted two months after whole-brain irradiation. Spatial memory preserved with >100 Gy/s dose rates, whereas it was lost after 10 Gy delivered with a conventional dose rate (0.1 Gy/s). Moreover, FLASH-RT yielded relative preservation of neurogenesis (retained 25% more subgranular neural stem cells) compared to CONV irradiation modality. A captivating aspect of this study is the researchers’ commitment to exploring the dose rate limits governing FLASH-induced neuroprotection. Consequently, they replicated the experiment with intermediate dose rates, resulting in a noteworthy decline in neurogenesis within the group irradiated at 30 Gy/s.

The outcomes prompted the same authors to publish a subsequent study in 2019 regarding the enduring neurocognitive advantages of FLASH radiotherapy (17). They employed the same prototype 6MeV electron beam linear accelerator (LINAC - Oriatron 6e) and administered three different doses (10, 12, and 14 Gy), only 12 and 14 Gy delivered with FLASH dose rate.

A diverse array of behavioral tests was utilized to assess memory and learning preservation one month after irradiation. The researchers furnished compelling evidence that FLASH-RT did not result in anomalies in the hippocampal or cortical regions of mice, except for the group exposed to 14 Gy.

Moreover, they validated their proposed radiobiological theories concerning oxygen depletion, free radicals, and inflammation using mouse and zebrafish models. Oxygen boost via carbogen abolished FLASH’s neuroprotection in mice, while zebrafish studies indicated that FLASH-RT generated a lower amount of toxic reactive oxygen species compared to CONV-RT, which could explain the reducing radiation-induced tissue damage. Specifically, zebrafish embryos preincubated with antioxidants were safeguarded from CONV RT injury in terms of body length, as compared to the FLASH-RT groups.

Regarding neuroinflammation, GFAP expression showed similar results in the 10 Gy FLASH group and non-irradiated controls at 14 days and 2 months post-treatment, respectively. FLASH also notably reduced activated microglia in the hippocampus a month after treatment. Lastly, FLASH was linked with the remarkable preservation of neuronal structure and dendritic spine density. An analysis of structural changes in hippocampal granule cell neurons conducted at one- and six-months post-exposure revealed that animals subjected to FLASH irradiation exhibited significantly higher numbers of dendritic spines (P < 0.05), amplified spine density (P < 0.0001), and increased spine volume (P < 0.01) at both post-irradiation time points. The same results were reported by Simmons et al. (18) following FLASH whole-brain irradiation with a customized clinical linear accelerator (Varian Clinac 21EX). As the most influential physical parameter for producing the FLASH biological effect remains uncertain, the focus of this study was on delivery time keeping beam parameters constant while altering pulse rate between FLASH and conventional methods. Neurotoxicity was evaluated through object recognition tests, showing reduced deficits after 30 Gy FLASH irradiation.

Further exploration of the neuroprotective potential was extended to juvenile mice exposed to a single dose of 8 Gy in 2020 (19). Alaghband et al. found that FLASH-RT mitigated radiation-induced cognitive dysfunction through various behavioral tests and persisted over time. UHDR irradiation protected developing and mature neurons (immature doublecortin+ neurons and mature double-positive labeled bromodeoxyuridine neuronal nuclei-Brd-NeuN), reduced microgliosis, and limited endocrine dysfunction (increased growth hormone).

In the same year, two additional studies were published, both involving female mouse models exposed to whole-brain irradiation (21, 22). On one hand, Allen et al. examined stroke risk from FLASH and CONV irradiation, assessing blood-brain barrier damage over time (24 hours, one week, one month). Doses of 25 Gy and 10 Gy with CONV (0.09 Gy/s) and FLASH (>106 Gy/s) dose rate explored early and long-term vascular toxicity. FLASH-RT reduced apoptosis in neurogenic brain areas (DG and SVZ) at the one-week mark, whereas it did not impact crucial vascular characteristics, such as blood vessel volume, eNOS expression, or tight junction proteins, unlike CONV irradiation.

On the other hand, Montay-Gruel et al. continued to study radiation-induced morphological and immunological changes, noting that pro-inflammatory markers C1q and C3 were elevated in both FLASH-RT and CONV-RT treated mice. Conversely astrogliosis (evaluation of hippocampal astrocytic morphology in terms of cell volume, thickness and length of dendritic processes) and immune signaling markers (GFAP, TLR4) were reduced in animals treated with 10 Gy FLASH-RT compared to those with CONV-RT.

#### 3.3.2 Hypofractionated regimen

Following the confirmation of the FLASH effect in a single dose, an attempt was made to demonstrate the continuity of the protective effect even when the radiation dose is fractionated, as is common in clinical practice.

Montay-Gruel et al. (41) studied anti-tumor efficacy and neuroprotective effect of FLASH-RT one month after exposure murine glioblastoma models, treated with different volumes and schedules of RT, including hypofractionated (4x3.5 Gy, 2x7 Gy, 3x10 Gy) and singular fractions (10, 14, 25 Gy). FLASH and CONV-RT equally hindered GBM growth, but only FLASH-RT improved cognitive issues after high doses and hypofractionated regimens.

Liljedahl et al. conducted a study on immunocompetent rats injected with NS1 glioblastoma cells, either subcutaneously or intracranially (33). Animals were exposed to two radiation fractions (8 Gy on days 8 and 14 for subcutaneous tumors, 12.5 Gy on days 9 and 13 for intracranial tumors) using CONV-RT or FLASH with a 10 MeV electron beam. No distinction was observed between these two methods in terms of tumor effectiveness, though the assessment of healthy tissue toxicity was absent. The study also affirmed the influence of tumor location on radioresistance and survival: mice with intracranial localization had a poor prognosis, with deaths before day 40 despite higher doses. Lastly, the researchers examined TIMP-1 protein, associated with growth and apoptosis, finding lower levels in animals with controlled tumors, aligning with extended survival in glioblastoma patients with low TIMP-1 expression.

The recent study of Limoli et al., focused on C57BL/6J female mice subjected to 30 Gy in 10 fractions, a standard-of-care fractionation regimen employed for treating multiple brain metastases (30).The aim of achieving effective intracranial control and cognitive preservation is essential in medical practice due to the potential neurological effects of whole-brain radiotherapy (WBRT) and the relatively lower intracranial control rate of stereotactic body radiation therapy (SBRT). Limoli et al. assessed the mice’s condition after 4 months, focusing on electrophysiological measurements of synaptic plasticity, particularly long-term potentiation (LTP). This study is the first to reveal the preservation of LTP with FLASH-RT in a fractionation scheme, implying that delivering WBRT at FLASH dose rates could effectively manage brain metastases while minimizing neurological toxicity compared to current practices.

Interestingly, the same research group had previously explored the hypofractionated scheme in mouse animal models and published two articles in 2022 and 2023 (29, 52). Male and female C57Bl/6 mice were divided into groups and exposed to hypofractionated whole-brain irradiation (2 × 10 Gy or 3 × 10 Gy with 48-hour intervals), either using FLASH-RT or CONV-RT, alongside unirradiated controls. After four months, cognitive status was assessed in the Object-Use in Later Test (OUL) in addition to the Novel Object Recognition (NOR) test and Light-Dark Box (LDB) arena, enhancing cognitive evaluation. FLASH-RT mitigated cognitive deficits induced by CONV-RT, maintaining synaptic plasticity, molecular markers, and structural components in multiple brain regions. It also reduced neuroinflammation and preserved cerebrovascular structure.

In conclusion, based on the analyzed studies, hypofractionated or monofractionated FLASH-RT induced effective neuroprotection compared with CONV-RT. Certainly, it is imperative to consider that the investigation of toxicity was conducted almost exclusively in healthy animal models. This choice was made to exclude potential complications arising from tumor presence and growth. While this approach may offer interference-free data, it overlooks the environmental context and associated reactions, potentially resulting in an unrealistic portrayal of radiation response. Therefore, future studies should prioritize investigating the response of healthy tissue in a more complex context such as the tumor microenviroinment.

### 3.4 Thorax

RT serves as the cornestone of treatment for patients with locally advanced non-small cell lung cancer (NSCLC), delivered concurrently or sequentially with chemotherapy. However, the prognosis remains unfavorable, primarily due to the inadequacy of CONV-RT in achieving effective loco-regional control of large size tumors. The RTOG 0617 study failed to demonstrate OS improvement though dose escalation in this setting of disease, likely attributed to cardiac and lung toxicity (53).Radiation-induced lung damage (RILI) is a significant dose-limiting factor in thoracic radiation. It can affect patients treated for lung cancer, breast cancer and lymphoma, with incidence ranging from 1% to 25% (54–57).

Two distinct phases characterize RILI : Radiation Pneumonitis (RP), an acute inflammatory condition of lung tissue, and Radiation Fibrosis (RF), a clinical ailment caused by persistent lung tissue destruction.

One of the initial in vivo experiments demonstrating the advantages of FLASH-RT over CONV-RT in reducing lung tissue damage was conducted by Favaudon et al. in 2014 (12). The authors investigated radiation-induced pulmonary fibrosis in C57BL/6J mice following bilateral thoracic exposure to a single dose 15- or 17-Gy CONV (dose-rate <0, 03 Gy/s) versus 17-Gy FLASH (ultrahigh dose-rate ≥ 40 Gy/s) irradiation, using a prototype linear electron accelerator capable of delivering 4.5 MeV electrons. Mice exposed to 15-Gy CONV showed initial signs of fibrogenesis, characterized by thickening and reorganization of the alveolar septa with collagen deposition, and inflammatory infiltration, 8 weeks post irradiation, that progressively worsening over time.

Additionally, the study detected that 30-Gy FLASH-RT induced fibrosis histologically comparable to that observed after 17 Gy CONV-RT. Favaudon’s study analyzed tumor progression too in a syngeneic orthotopic tumor model, consisting of TC-1 cell engineered to express luciferase (TC-1 Luc+) and orthotopically implanted in the lungs of C57BL/6J mice. The study demonstrated that 15-Gy FLASH was as effective against the tumor as 15-Gy CONV. Furthermore, a dose escalation study showed that the 28-Gy FLASH dose was significantly more effective against tumor growth. In fact, 80% of mice irradiated with 28-Gy FLASH were still alive, and 70% of them were free of tumors 62 days post-irradiation, with no signs of fibrosis.

In 2020, Fouillade et al. revealed that FLASH minimized the generation of pro-inflammatory genes and DNA damage in normal tissue, spares lung progenitor cells from excessive damage and reduced the risk of replicative senescence (36). They utilized C57BL/6J wild type and Terc-/- mice exposed to bilateral thoracic irradiation using a 4.5-MeV linear electron accelerator in both FLASH and CONV modes. Lungs exposed to CONV-RT showed a double increase in the number of proliferating cells after one-week post-irradiation compared to non-irradiated controls (8% vs 4%), instead the number of proliferation cells in the parenchymal lung exposed to FLASH irradiation was not significantly superior to those of the control. The sc-RNAseq analysis of lung cells 4-days post-irradiation demonstrated that FLASH-RT induced a lower expression of inflammatory genes compared to CONV-RT.

By immunofluorescence, the authors assessed the persistent 53bp1 foci, markers of cell senescence, in lung cell isolated after 1 week and 3 months from irradiation as well as in control non irradiated lung cells (58, 59). After one week, both CONV and FLASH irradiated lungs showed a high number of cells with 53bp1 foci, with a higher number of foci per cell observed after CONV irradiation. After three months, the number of foci of 53bp1 per cell decreased in FLASH-irradiated lungs but increased in CONV-irradiated lungs, suggesting that DNA damage continues to accumulate over time. Furthermore, the expression of other senescence markers, such asSASP (Secreted Associated Senescence Proteins), Cdkn2a, Serpine1 and Mmp-2 was relatively lower following FLASH-RT than CONV irradiation at three- and five-months post-irradiations.

In 2020, Kim et al. (32) directed their attention to investigating the biological mechanisms underlying FLASH-RT (total dose 15 Gy) in lung cancer cells (Lewis lung carcinoma LLC) inoculated into mouse models (male C57BL/6). Interestingly, the authors observed that FLASH-RT tumor cells had an increase intracellular ROS level determined by DCFDA staining but lower γH2AX+ levels at 6 hours post-irradiation. To explain this contrasting data, the authors supposed that FLASH-RT might produce more ROS in the cytosol than in the nucleus and/or that is associated with a very late formation and a fast repair of DNA double strand breaks. Moreover, the authors inoculated tumor cells in the subcutaneous tissue of mice. Following cutaneous irradiation there was a rapid vascular collapse with FLASH-RT but not with CONV RT, which highlighted the protective effect of FLASH-RT on the vasculature. They noticed contracted vessel morphology at 6 hours post-CONV irradiation compared to controls, but this difference disappeared at 48 hours post-irradiation. Notably, no difference in contracted vessel morphology was observed under FLASH irradiation compared to controls at either 6 or 48 hours, suggesting that rapid and reversible vascular collapse did not occur with FLASH-RT.

The ubiquitous representation of the vascular system in the body implies that the analysis of endothelial damage can be considered across all anatomical regions. Indeed, the damage of these cells represents a pivotal event in the initiation of various processes, including the mechanisms of skin and lung fibrosis, as well as brain radionecrosis.

### 3.5 Abdomen

Radiotherapy is widely used for treating tumors in the abdominal and pelvic area. For instance, concomitant platinum-based chemo-radiotherapy with external beams plus intrauterine brachytherapy is the standard of care for locally advanced cervical cancer, concomitant 5-FU based chemo-radiotherapy is commonly used as neoadjuvant preoperative treatment in rectal cancer, and radiotherapy can be sometimes used as adjuvant post-surgical therapy or palliative treatment in pancreatic cancer.

Radiotherapy of these tumors is associated with early and late side effects. In fact, diarrhea, nausea, vomiting, inappetence, cramping and abdominal pain may occur due to irritation of the gastrointestinal system by ionizing irradiations. The risk of small bowel perforation severely restricts the doses that may be delivered to large tumors strictly close to these healthy tissues.

Similarly, the management of prostatic cancer is impacted by this factor, thereby invalidating treatment efficacy and patients’quality of life. In gastrointestinal malignancies, such as pancreatic cancer, the disease’s aggressiveness along with radiation induced toxicity relegates RT to a peripheral role in the management of patients afflicted with this condition. This has led to an investigation of the effect of FLASH-RT on healthy tissues in the abdominal district, in order to overcome this barrier.

Loo et al. (14) in 2017 were the first that documented a significantly increased survival after FLASH abdominal irradiation of mice. Total abdominal irradiation was administered on male C57BL/6 mice at doses ranging from 10 to 22 Gy using a clinical linear accelerator with a 20 MeV electron beam, while comparing dose rates of 0.05- Gy/s (CONV) or 70- and 210-Gy/s (FLASH). Survival rates 20 days post-irradiation was 29% in mice treated with CONV-RT compared to 90% for those treated with FLASH-RT.

Venlkatesulu et al. observed in 2019 (16) that UHDR -RT (35 Gy/s) caused more gastrointestinal mucosal toxicity than CONV irradiation. To demonstrate the effects on the gastrointestinal mucosa, BALB/c mice were exposed to a single 16 Gy fraction of whole abdominal radiation. Mice exposed to CONV irradiation survived until day 15 while all FLASH-treated mice died within 7 days. Therefore, normal tissue sparing in FLASH irradiation is not universal and may depend on a number of additional but unknown biological factors and/or treatment parameters.

Ruan et al. (26) demonstrated that FLASH-RT can spare mouse intestinal crypts and had a lower impact on gut microbiome composition. Female C3H mice received CONV-RT (average dose rate 15 Gy/min = 0.25 Gy/s, dose-per-pulse ≈ 10 mGy, pulse dose rate ≈ 3×103 Gy/s) or FLASH-RT (doses ranging from 7.5 to 20 Gy =2.2 to 5.9 × 106 Gy/s). This latter was associated with a significantly lower crypt damage and a less microbiome alteration. A statistically significant difference was found in crypt survival for FLASH-irradiated mice at doses from 7.5 to 12.5 Gy (the dose to reach 10% remaining crypts for CONV irradiation was 12.7 Gy and for FLASH it was 13.9 Gy).

Microbial diversity revealed that the cluster of FLASH-irradiated mice were closer to the cluster of non-irradiated mice, indicating less microbiome alteration than the exposed group to CONV irradiation. The intestinal epithelium is more damaged with CONV RT compared to UHDR with consequent bacterial translocation. This results in activation of an inflammatory response that induces an alteration of composition of the microbiome. FLASH RT group seems to spare the intestinal mucosa and subsequently preserve microbiome composition.

Given the importance of the correlation between the physical parameters of the beam and the flash effect, the authors decided to test the FLASH effect by varying the pulse structure and the time interval between pulse administration. They have demonstrated that increasing the number of pulses or the time interval between double-pulse administration gradually increased normal tissue toxicity and thus decreased the FLASH effect. Overall, they documented that the normal tissue-sparing effect of FLASH irradiation was correlated with the average dose rate and time pulse structure.

In contrast to Venkatesulu’s results, the following year Levy et al. (23) showed a reduction in radio-induced intestinal lesions, preserving intestinal function and epithelial integrity. This resulted in lower mortality rate due to gastrointestinal syndrome compared to CONV irradiation. Female C57BL/6 mice were irradiated over the whole abdominal cavity with 16 Gy in FLASH (216 Gy/s) or CONV (0.079 Gy/s)RT. A modified clinical linear accelerator was used to generate a 16 MeV electron beam and to deliver a homogenous depth dose (within < 10% heterogeneity).At a check 8 days post irradiation, mice lost an average of 26-30% body weight in both irradiation modalities. In the FLASH mode, 90% of the irradiated mice recovered their original body weight and survived more than 90 days after irradiation. In contrast, mice irradiated in the CONV mode continued to lose body weight and died within 10 days.

The histological analysis of jejunum demonstrated that FLASH mode-irradiated mice had an increase in the number of regenerated crypts at 96 hours post-irradiation compared to CONV-irradiated mice. Furthermore, the histological analysis after 12 weeks of FLASH irradiation was indistinguishable from control animals. Moreover, the authors observed an initial decrease in the regeneration of intestinal crypts (expressed by the number of BrdU+ cells per crypt) from 4-72 hours post irradiation in the CONV group and from 4-48 hours in the FLASH group; regenerated BrdU+ crypts started appearing 96 hours after irradiation in the CONV group and 72 hours in the FLASH group. These results suggested that crypt regeneration is stronger after FLASH irradiation compared to CONV. In addition, they demonstrated that abdominal FLASH-RT preserves crypt base columnar cell (CBC) proliferation compared to CONV-RT, indicating that intestinal stem cells can be spared from death after FLASH-RT.

To understand how the FLASH mode spared radiation-induced cell death, they quantified the number of γ-H2AX+ in intestinal CBC cells of mice treated with total abdominal irradiation with 14 Gy in FLASH and CONV modes. They found that there was a modest reduction in initial double-stranded DNA breaks of intestinal CBC cells in mice after FLASH irradiation, with a consequent increase in damage repair.

Regarding tumoral control, two studies (23, 34) had compared the efficacy and safety between FLASH and CONV-RT using C57BL/6 mice after intraperitoneal inoculation of ID8 ovarian cancer cells. Levy et al. (23) analyzed the total tumor burden, finding a decrease in the number of tumor nodules and total tumor weight in irradiated mice compared to controls, without finding a significant difference comparing the irradiated mice with FLASH and CONV mode. Therefore, FLASH and CONV-RT appear to have similar efficacy in reducing the tumor burden of ovarian cancer in the peritoneal cavity of mice, suggesting that FLASH irradiation may be an effective strategy to improve the therapeutic index of radiotherapy for abdominal-pelvic tumors.

In a preclinical mouse model of ovarian cancer, Eggold et al. (34), have confirmed that FLASH-RT at the abdominal-pelvic level promoted intestinal regeneration and maintained tumor control. Since many effects of FLASH irradiation still remain unknown, they investigated the immunomodulatory effects of total abdominopelvic irradiation with CONV and FLASH modes using female C57BL/6 mice. The mice were irradiated 10 days after intraperitoneal inoculation of ID8 or UPK10. After FLASH-RT, the mice showed an increased number of regenerated crypts compared to CONV-RT. In addition, at day 27 post irradiation there was a reduction in tumor and ascites in mice irradiated in both modalities compared to controls, with no significant difference between the two types of irradiations.

In the analysis of the immune environment 96 hours after irradiation the authors found a reduction of DC45+ leukocytes and T and B cells, with a shift in the ratio of T cells in the tumor microenvironment in both irradiation modalities compared to non-irradiated. Furthermore, they detected an increase of CD4+ cells in the tumor microenvironment in mice exposed to FLASH irradiation compared to those treated with CONV. Subsequently, 17 days post irradiation, mice exposed to CONV or FLASH irradiation showed enhancement of CD107a+ and CD8+ T cells.

Next, the researchers randomized 6 cohorts to study the immunomodulatory properties of abdominopelvic irradiation in the ID8 synergistic model of ovarian cancer: isotype control antibody (IgG), IgG + 14 Gy CONV, IgG + 14 Gy FLASH, αPD-1, αPD-1 + 14 Gy CONV, or αPD-1 + 14 Gy FLASH. After 27 days of the injection, in the arms exposed to irradiation (FLASH or CONV) + IgG and αPD-1, a reduction of tumor weight and ascites was found. However, they observed that the combination of FLASH + αPD-1 had a higher efficacy than FLASH + IgG.

In the groups that had the combination of CONV or FLASH irradiation with αPD-1, they showed an increase in tumor infiltrating CD8+ cells and reduced the immunosuppressive Neutrophils and polymorphonucler myeloid-derived suppressor cells (PMN-MDSC) and M2 to M1 macrophage ratios in the tumor microenvironment. The study showed that FLASH irradiation associated with αPD-1 is effective in tumor control and improves intratumoral infiltration of CD8+ T cells, reducing immunosuppressive monocytes in the ID8 model resistant to αPD-1.

## 4 Discussion

The current literature regarding electron UHDR strongly supports the existence of FLASH effect. The evidence of a potential protection on healthy tissues of UHDR RT has roots dating back to the 1960s-1980s. For years, the concept of enhancing radiotherapy through FLASH dose delivery has remained dormant until, driven by technological advancements, researchers took up the studies again.

As evidenced by the analysis conducted in this review, numerous studies have delved into radio-induced toxicity in animal models, exploring various aspects in detail. While initial in vivo studies primarily centered around the skin, confirmations have also emerged in other anatomical regions, including thorax, nervous central system, head and neck, and abdomen.

It appears that UHDR causes less damage to stem cells compared to CONV-RT. This conclusion stems from studies involving neuronal, hematopoietic, intestinal, and cutaneous stem cells. Moreover, mature cells also seem to be spared from radio-induced damage under UHDR regime. This translates into a lesser severity and duration of acute and late tissue damage. Clinically, improvement in memory, preservation of intestinal and pulmonary organ functionality, and ultimately a lower incidence of grade 3-4 skin reactions has been demonstrated. Furthermore, these experiments contributed significantly to the investigation of the radiobiological mechanisms underlying the phenomenon, although clear answers remain elusive to date.

The oxygen depletion theory, which has been long considered, has been recently criticized. In fact, by measuring oxygen concentration in pure water after FLASH-RT, Jansen et al. (60) demonstrated that UHDR does not consume all the oxygen present in solution.

Other theories took into consideration the difference of free radical concentration between cancer and normal cells after FLASH RT and CONV-RT, respectively. The higher difference detected following CONV-RT could reflect a diversity in peroxidized compounds metabolism, labile iron concentrations and radical self recombination mechanism between cancer and healthy tissues (61).

Another appealing hypothesis concerns a different impact on the immune system. Following IR exposure, immune cells release several proinflammatory cytokines, including TNF- a, IL-6, IL10, that increase the damage of surrounding healthy tissue. In vivo studies seem to show that FLASH-RT causes a decreased inflammatory cell activation, which might have a role in healthy tissue sparing.

Moreover, CONV- RT induces a depletion of both immune mature cells and immune stem cells, whereas FLASH RT seems to spare both these immune cell types, thus preserving immune system anticancer activity. The possible interaction between FLASH-RT and immune checkpoint inhibitor administration could represent a very promising field of translation and clinical research (62).

### 4.1 Flash experimental question

The definition and characterization of the optimal dose rate(s) able to produce the FLASH effect represent an active topic of research. From early studies of 70’s it is clear that irradiation at UHDR represents the hallmark of FLASH-RT delivery. However, recently published papers have emphasized the relevance of additional physics parameters such as the instantaneous dose rate (IDR), dose-per- pulse, pulse frequency and pulse duration (63).

It is still debated which are the most important beam parameters related to the Flash effect and their quantitative dependence on this phenomenon. In most studies conducted to date, only three parameters (Ḋ_m,_ DPP, IDR) have been set on all but the possible combinations remaining to be tested are countless. This has not been possible due to electron LINAC used for experiments that could not vary all the beam parameters linked to the FLASH effect (64). Furthermore, most of these studies (15, 31) have used modified medical and industrial linacs to achieve UHDR, mostly by removing important components from the beam path such as the monitor chambers (65).

Beam monitoring, which is fundamental for clinical LINAC, is performed through an ionization chamber to ensure that the real-time dose delivered matches with what has been planned. However, the commonly available ionization chambers experience a process of saturation with UHDR pulsed beams, and therefore the early experiments with UHDR did not have a beam monitor system able to assure the right beam erogation (66–68).

This problem has been solved through the implementation of a new type of beam monitoring based on the non-invasive measure of the electrons fluence at the exit window level by means of a current transformer system. This is a passive monitoring device able to reliably measure dose rate without saturating and without perturbing the beam fluence at UHDR. This innovative system has been used only in the recent studies aimed to investigate Flash effect UHDR pulsed electron Linac (69–71).

Additionally, dosimetry has been mainly performed with passive dosimeters such as radiochromic films, while active online dosimetry has not yet been used due to significant saturation issues (69, 72). The dosimetric analysis of the total dose delivered has been extensively performed with GafChromic film. Its detection principle relies on radiation-induced polymerization of an active (diacetylene) layer, resulting in a rise of optical density (OD). GafChromic film has been employed in Flash studies for its excellent spatial resolution, for its energy and dose rate independence. However, it measures the dose delivered offline, generally 24 hours after exposure (73).

A new generation of dosimeters for UHDR (such as Silicon diodes, MOSFETS and Semiconductor detectors) has been produced. These novel tools are able to adequately measure the target dose online (74). The availability of research dedicated linacs, such as the Sordina IORT Technologies S.p.A. (SIT) ElectronFlash (75), which guarantee a reliable beam delivery and real time beam monitoring through AC current transformers (ACCT) as well as active dosimetry solutions (66, 70, 71, 76–78), can significantly improve the biological and clinical research in the field of electron FLASH-RT. Moreover, the evaluation of dose distribution is another critical aspect of RT, since in vivo irradiation of inhomogeneous tissues with charged particles does not produce homogeneous dose-distribution. Still the currently available FLASH studies lack an imaging system and treatment planning system able to optimize and calculate the depth dose distribution.

The main limitation of the use of electrons is the low depth penetration into tissues, limited to a few centimeters. In fact, to date the only human trial for the treatment of deep tumors with FLASH RT (symptomatic bone metastase-FAST-01) employed proton particles (79). Nevertheless, electrons are more flexible and can easily reach high DPP values necessary to trigger the flash effect compared to protons. Franciosini et al. have shown with Monte Carlo simulations that an energy of 100-250 MeV, multiple fields and a pencil beam scan are required to obtain satisfactory dosimetric conformations in cases of deep tumors by using fields of few mm (80). For this reason, new linear accelerators able to deliver UHDR with high-energy electrons (VHHE) must be developed in the future for these treatments.

Novel in vivo and in vitro experiments must be conducted in order to understand if FLASH effect is preserved with pencil beam irradiation modality and with using multiple fields. All these additional investigations are strongly warranted before FLASH RT can be employed in the clinical practice.

**Figure 2:**
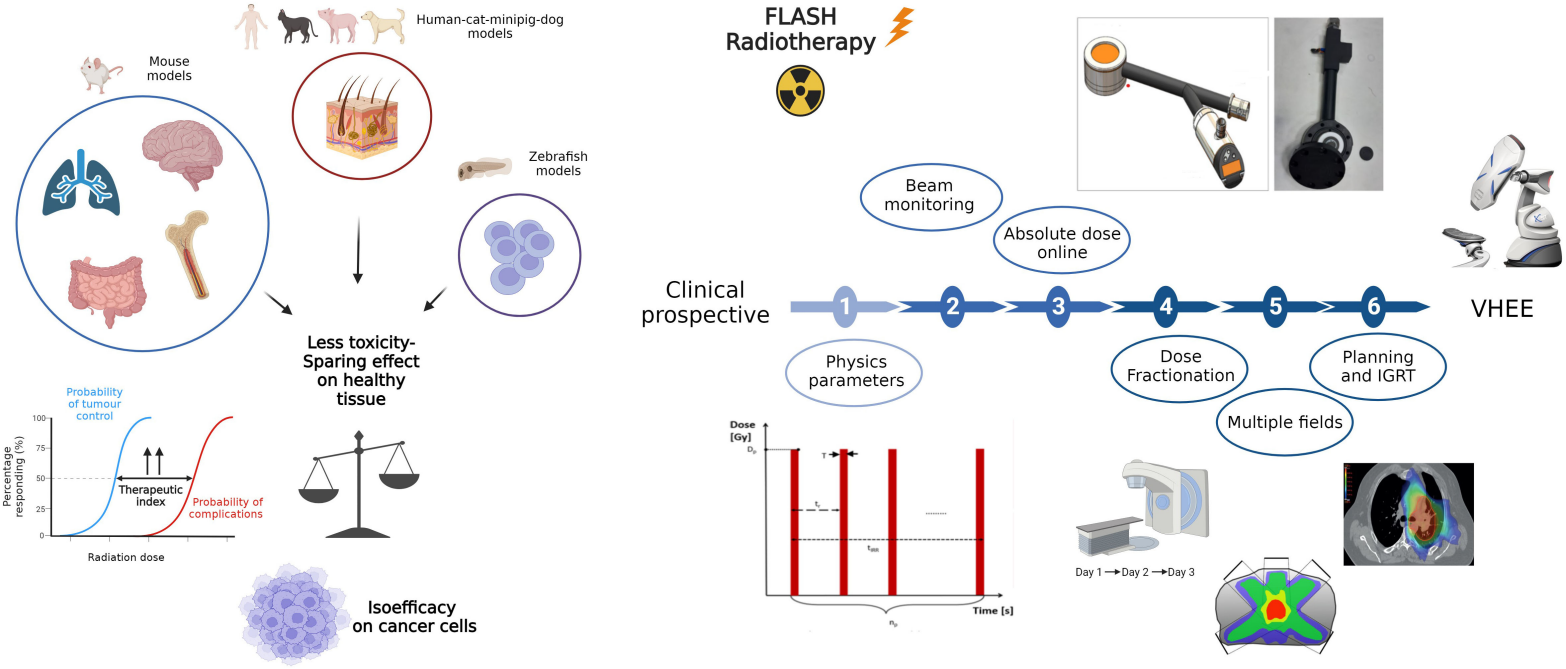
A graphic representation of in vivo preclinical studies. The oblstacles to overcome in order to apply FLASH in clinical setting are illustrated on the right. In details, it involves determining the physical parameters to trigger the FLASH effect and the relationship between phisycal parameters and biological mechanisms, resolving technological issues in beam delivery and monitoring, confirming the effect with fractionation, large volumes and multiple fields, understanding if the time lapse to pass from one to another field could compromise the FLASH effect and optimizing dose distribution. Created with BioRender.com.

In conclusion, several questions are still unsolved (**Figure 2**), such as:

i) the impact of different physical parameters on the FLASH effect
ii) the possible impact of dose fractionation on FLASH effect
iii) the correlation of FLASH effect with the spatial distribution of the dose

### 4.2 Clinical prospective

Low energy electrons have been employed in radiotherapy since the early 1950s. In clinical practice, these particles are used to treat superficial or semi-deep tumors extended to the surface of the skin and in the intraoperative radiotherapy (IORT) setting. This is related to the low penetration power of electrons in tissues. Compared to X-rays, electron dose falls off rapidly, allowing the target to cover within a few centimeters from the surface. In fact, the first field of clinical application of FLASH RT has been superficial tumors (cutaneous lymphoma). This choice is linked to several technical advantages. The treatment requires the delivery of dose on a single field with a simple set-up and without a planning system.

Currently, skin cancers are usually treated with surgery, eventually followed by adjuvant RT in presence of risk factors on surgical samples, or with primary radiotherapy when the lesion cannot be radically removed. FLASH RT may improve local control through dose escalation with better cosmetic results.

Two clinical trials are currently enrolling patients for treatment of skin cancer malignancy with electron FLASH RT. The IMPulse trial, a phase I dose escalation study of FLASH therapy in patients with cutaneous metastases of melanoma and the LANCE trial, a Phase II study for Patients With Localized Squamous Cell Carcinoma or Basal Cell Carcinoma. Low energy electron should be also tested in IORT, a radiotherapy technique sometimes used for selected cases of patients with recurrent soft tissue sarcoma, rectal and cervical cancer (81–83). It has been also employed in adjuvant therapy in breast and pancreatic cancer surgery alone or combined with external-beam radiation therapy (84, 85).

FLASH IORT may improve therapeutic index reducing the risk of severe events, such as intestinal perforation, vascular damage, wound complications, anastomotic leakage, bladder dysfunction and neuropathy.

With the future development of VHEE, it might be also possible to treat deep tumors. Such as radioresistant tumors like glioblastoma or large cancer arising in “parallel” organs like stage III NSCLC. VHEE may also be useful in the treatment of radiosensitive tumors like HPV+ SCC of head and neck in which radiotherapy already offers good local control but with severe late effects such as dysphagia and xerostomia. Finally, FLASH VHHE may play a major role for the treatment of tumor recurrence in previously irradiated high-dose regions.

## 5 Conclusion

In several experimental model, FLASH RT has shown the same efficacy as CONV RT against different types of cancer, such as squamous cell carcinoma, ovarian cancer, lung cancer, glioblastoma, CD30+T-cell cutaneous lymphoma, associated with a significant low damage of surrounding normal tissue. Although the evidence highlights this benefit on healthy tissues, the path toward clinical application remains lengthy and challenging, hinging on achieving systematic reproducibility of the phenomenon.

While research must delve into the radiobiological mechanisms triggered by UHDR irradiation, understanding how to induce FLASH effect from the perspective of beam characteristics is equally crucial. The in vivo experiment seems to indicate that a dose rate >40 Gy/s with a total irradiation time < 200 ms must be reached (**Tables 1**, **2**). However, it is still debated which are the most important beam parameters related to the FLASH effect.

New experiments with novel dedicated technologies must be performed to better understand the physical aspects related to this phenomenon in order to use electron FLASH RT in clinical practice.

In the future FLASH RT, especially with the development of VHEE, could be useful to treat radioresistant tumors, large sized tumors, and previously irradiated neoplastic lesions (86).

## Data availability statement

The original contributions presented in the study are included in the article/supplementary material. Further inquiries can be directed to the corresponding authors.

## Author contributions

NG: Writing – original draft, Writing – review & editing. GG: Writing – original draft, Writing – review & editing. TF: Writing – original draft, Writing – review & editing. AG: Methodology, Supervision, Writing – review & editing, Writing – original draft. FD: Writing – review & editing, Writing – original draft. PP: Conceptualization, Visualization, Writing – review & editing. MN: Writing – review & editing. FrP: Writing – review & editing. SC: Writing – review & editing. FaP: Funding acquisition, Supervision, Validation, Writing – review & editing.
